# A Blended Educational Program to Promote Dialogue on Patient Safety Between Patient and Family Advisory Councils and Health Care Organizations: Codevelopment Study

**DOI:** 10.2196/79286

**Published:** 2025-11-24

**Authors:** Yannick Blum, Larissa Brust, Matthias Weigl, Qëndresa Rramani Dervishi

**Affiliations:** 1 Institute for Patient Safety, Medical Faculty, University Hospital Bonn, Venusberg-Campus 1, Bonn, 53127, Germany, 49 22828713881

**Keywords:** patient safety, communication, collaboration, patient engagement, educational program

## Abstract

**Background:**

Reducing patient harm and improving patient safety is a central objective in global health care. Effective communication and meaningful patient engagement are considered essential strategies to achieve this goal. However, implementation of structured and strategic patient engagement at the organizational level remains limited, particularly in the context of patient safety. Patient and family advisory councils (PFACs) offer a promising model to enhance organizational-level patient engagement, yet guidance on implementation and targeted training for PFAC members is scarce.

**Objective:**

This study aimed to codevelop an evidence-informed, blended educational program designed to strengthen PFAC members’ competencies in patient safety and communication, and to foster strategic collaboration between PFACs and health care organizations.

**Methods:**

The intervention was systematically developed using a logic model framework that structures the development process from available and required resources to the ultimate objectives and impacts. The primary target group included PFAC members, such as patients, relatives, and advocates, as well as health care representatives in leadership, quality management, or coordination roles. The program’s content and structure were informed by a nationwide needs and requirements analysis among PFAC members, conducted using a mixed methods Delphi approach, and by a rapid scoping review on existing educational resources and evidence on PFAC engagement in patient safety.

**Results:**

Our *Partners for Patient Safety* blended educational program consisted of 2 modular components: a self-paced e-learning module and a subsequent on-site workshop module. Content addressed three core topics: (1) fundamentals of patient safety, (2) engagement of PFACs, and (3) communication and collaborative goal setting. The e-learning module provided theoretical knowledge using diverse didactic formats, such as interactive tasks, videos, and downloadable materials, and included applied examples using established decision-making and goal-setting frameworks. The workshop module built on the e-learning module and facilitated local implementation through collaborative exercises focused on stakeholder perspectives, communication barriers, and joint goal development. Both modules were aligned with defined learning objectives and combined passive and active learning strategies to promote engagement and practical application.

**Conclusions:**

The *Partners for Patient Safety* program seeks to develop PFAC members’ competencies, promote collaboration in patient safety, and foster a culture of safety and partnership within health care organizations. By combining theoretical knowledge with practical, collaborative learning, the program addresses key barriers to effective PFAC engagement at the organizational level. Its modular design allows flexible implementation and has the potential to strengthen cooperation between PFACs and health care representatives, ultimately improving patient safety outcomes. Further evaluation of the program’s implementation and effectiveness is needed.

## Introduction

Reducing patient harm and improving patient safety is a central goal in global health care, as emphasized by the World Health Organization [1]. Effective communication within health care organizations and improved patient engagement are essential strategies for achieving this objective [1-3]. Nonetheless, respective approaches to foster effective patient engagement for strategic promotion of patient safety on the level of health care organizations are rarely reported [4]. Thus, effective and evidence-based strategies for successfully implementing patient engagement at the organizational level are greatly needed.

Patient engagement refers to involving patients and their representatives in a patient’s health care journey, potentially taking place at all levels of care; it can occur at all levels of care and range from consultation to partnership [5]. Despite widespread advocacy in research and policy for increased meaningful patient engagement across all levels of health care [6,7], in reality, its implementation remains limited, especially in the context of patient safety [8-10]. These shortcomings are particularly pronounced at the organizational level, where strategic engagement of patients and their representatives faces several barriers [10]. One promising approach to increase patient engagement at the organizational level is the establishment and integration of patient and family advisory councils (PFACs) in health care organizations [11,12]. PFACs, or “patient advisory boards,” are often constituents of patients and their families, as well as members of other patient representative groups (eg, self-help groups), who might also be engaged in existent processes such as quality improvement and patient safety [11].

Despite a few general recommendations on implementation [13,14], practice guidelines and standards regarding implementation of patient engagement at the organizational level are rare [11,15]. While available research suggests that engagement of advisors in safety and quality improvement initiatives yields positive outcomes [16,17], our previous rapid scoping review found that hands-on recommendations or practice standards for advisors assuming this role are mostly lacking (reported elsewhere). This gap persists despite long-standing calls from patient representatives for more training and education on patient safety topics [18]. Moreover, a previous survey conducted in the German health care setting revealed that levels of patient engagement and patient advocacy are perceived as low, partly attributed to a shortage of respective educational programs for patients and their representatives [10]. Addressing these persistent needs requires targeted interventions to enhance engagement and skills of patient representatives such as those within PFACs.

To this end, we codeveloped a blended educational program on relevant topics on patient safety and communication for PFACs and associated health care representatives, drawing upon needs of PFAC members and with continuous support of patient representatives. This intervention program will strengthen patient safety and communication skills of PFAC members and increase patient engagement at the organizational level of health care organizations, thereby promoting patient safety.

Our main objective was to describe the process of codevelopment, the framework, and content of the *Partners for Patient Safety* (P4PS) program for PFACs and associated health care representatives. This paper presents the underlying evidence and provides a detailed overview of the structure, methods, and components of the intervention.

## Methods

### Overview

This section covers (1) the systematic development process; (2) the evidence base of previous extensive work, specifically a needs and requirements analysis and a rapid scoping review (reported elsewhere in detail) [19]; and (3) the setting and target group of the P4PS program.

We used the Template for Intervention Description and Replication (TIDieR; Checklist 1) and the Guidance for the Reporting of Intervention Development (GUIDED; Checklist 2 ); both frameworks ensure comprehensive reporting of the intervention [20] and enhance its reproducibility [21,22].

### Ethical Considerations

The study was preregistered in the German Clinical Trials Registry (DRKS00034733) and approved by the ethics committee of the medical faculty at the University of Bonn (2024‐236-BO). Data collection and processing were carried out in accordance with the Declaration of Helsinki, the European Union’s General Data Protection Regulation, and the German Federal Data Protection Act. The privacy and confidentiality of all participants were strictly maintained; all identifying information was removed or anonymized before data analysis. All participants signed an informed consent form and received a monetary compensation of €120 (US $1.16) for their participation.

### Intervention Development

#### Intervention Framework

**Figure 1.**
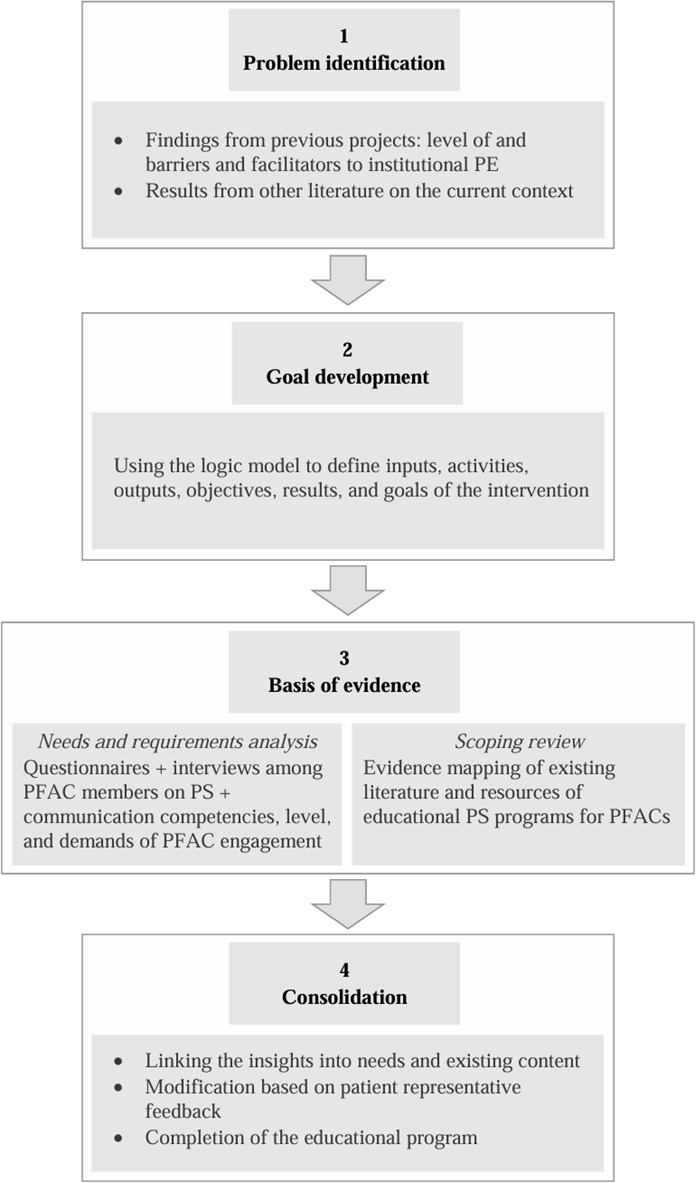
Process of developing the Partners for Patient Safety program. PE: patient engagement; PFAC: patient and family advisory council; PS: patient safety.

For systematic development of the intervention, we used the logic model [23] as a structured framework and well-established tool in public health and quality improvement research, facilitating the systematic planning, implementation, and evaluation of interventions by mapping the relationships between inputs, activities, outputs, objectives, results, and goals [24,25]. Specifically, the model guided the derivation of necessary inputs to promote exchange and collaboration between the PFAC and the health care organization through the development of skill acquisition in terms of patient safety and communication among learners. The details of the model were developed iteratively by 2 members of the research team based on problem identification and objectives of the intervention. Figure 1 shows the general process and procedure for developing the P4PS program. This methodological approach ensured a goal-oriented intervention design based on key assumptions and evidence. An overview of the columns of the logic model relevant to the process of intervention development is provided in Table 1. The complete model can be found in Multimedia Appendix 1.

**Table 1.** Logic model for Partners for Patient Safety program development. The table outlines the levels, descriptions, and key assumptions of the logic model. The vertical logic works backward from inputs to goals to establish a conceptual framework and operational definitions for each level, while the horizontal logic describes the requirements needed to achieve each level. The columns objective verifiable indicators and means of verification of the model are intended for evaluation purposes and are therefore not included here.

Level	Description	Key assumptions
Goal	Improving PS^a^ and quality of care at an organizational level by engaging patients, their families, and representatives in health care	Increased engagement of PFACs^b^ in health care in Germany improves PS Engagement in PS is hindered by inadequate knowledge of PFAC members regarding PS and communication
Results	Increased PFAC engagement in decisions on quality, care processes, and PS issues Sustainable networks and partnerships between PFACs and HCO^c^	PFACs and HCRs^d^ are interested in promoting PFAC engagement in PS topics PFACs and HCO know how PS-relevant topics can be evaluated
Objectives	Increased engagement of PFACs through improved quality and quantity of communication between participating PFACs and HCR on PS topics	PFACs need better PS and dialogue skills for gaining a competent voice in health care Communication and partnership-based dialogue between PFACs and HCR must be expanded
Outputs	Participating PFAC and HCR Understand the essential principles of PS and recognize events relevant to PS Understand the models and strategies of effective communication presented Understand examples of good PFAC engagement in care processes Understand the perspectives of various stakeholders and explain their activities and functions Identify barriers and misunderstandings in communication between PFAC and HCO Develop new possibilities for future activities and collaboration	Participating PFAC and HCO Are interested in promoting PFAC engagement in PS processes Are interested in discussing and reflecting on cases of (near) harm that have occurred in patient care Speak openly about communication barriers and PS risks Additionally to the above HCOs provide opportunities for closer collaboration Resources are made available to ensure improved collaboration
Activities	Intervention development based on needs and requirements as well as international literature PFAC and HCR Are trained in PS content, examples of PFAC engagement, and communication skills Are presented with new communication activities to foster dialogue Are given the opportunity to systematically apply the options presented to their own situation Receive support to promote common goals and future collaboration	PFAC and HCR take part in surveys and interventions PFACs share specific needs and requirements on PS and dialogue topics Interventions developed are effective in promoting PS and communication skills Interventions developed are effective in promoting collaboration
Inputs	Interview guide for needs and requirements analysis Online questionnaire to prioritize content for intervention Literature research on comparable, already existing programs Other infrastructure and resources, for example, Funding Personnel expertise of study team Support with and resources for the development of educational programs	Responsible people provide Resources to develop the intervention Materials for the participants Generally existing Adequate funding Availability of PFAC and HCR Willingness and consent of PFACs and HCR to participate No ethical concerns about the project

a PS: patient safety.

b PFAC: patient and family advisory council.

c HCO: health care organization.

d HCR: health care representative.

#### Target Population

The target group of the P4PS program comprised members of PFACs affiliated with health care organizations. This included representatives of patient-oriented interests (eg, patients, relatives, patient advocates, and representatives of other patient-related organizations) as well as professionals of the health care organization who were responsible for leadership, general management, or administration of the respective PFAC (eg, coordinators and moderators). In line with the intervention’s objective of promoting collaboration between PFACs and health care organizations, program development was additionally geared toward the participation of senior professionals and leaders from collaborating health care organizations, such as senior physicians and head nurses, quality and risk managers, or members of hospital management and board of directors.

#### Evidence to Inform Development of the Intervention

Two previous main sources informed the development of the P4PS program in terms of topics and formats: a needs and requirement analysis (a mixed methods study with a Delphi approach) and a literature review on existing educational resources for PFACs; both are reported elsewhere in detail [19]. However, a brief summary of the key findings from both studies is provided in Multimedia Appendix 2 [19].

The mixed methods study with a Delphi approach previously identified PFACs’ needs regarding competencies in patient safety and communication as well as PFAC engagement [19]. This approach ensured a high degree of participation in informing and developing this intervention. According to the continuum of patient engagement outlined by Carman et al [5], it actually achieved “active involvement” of PFAC members. According to the stages of participation described by Wright [26], the degree of participation in this study can be classified as medium, meaning participants had partial decision-making authority. A total of 19 participants from 6 nationwide PFACs took part in the two rounds of surveys, which consisted of (1) interviews and (2) questionnaires. The responses were evaluated descriptively and consensus-oriented (criterion: 85% agreement). The mixed methods analysis was performed sequentially and convergently. Among other findings, the results highlighted the need to address the following: (1) fundamentals of patient safety, error occurrence and error prevention, and knowledge on quality and risk management; (2) support in understanding and defining PFAC roles, responsibilities, and tasks; and (3) precise and clear communication of information, complaints, and problems. In terms of formats of intervention, participants desired a modularized program with theoretical and practical components, delivered online or on-site depending on the topics and organization, with opportunities for partial implementation in their respective settings [19].

To further inform the development of the P4PS program, we previously used peer-reviewed and relevant gray literature from various databases and search engines to conduct a parallel literature search in the form of a rapid scoping review of existing evidence and educational resources on patient safety for PFACs in health care organizations (reported elsewhere in detail). Although the number of the identified sources was limited, 13 articles and sources featuring various formats, content, and topics could be identified [16,27-38]. This information was extracted, and, together with the results of the needs and requirement analysis, used to inform the content and format of the intervention. In addition, other sources, such as previous projects and teaching materials, as well as those from various organizations, were used to generate intervention content [1,39-43].

While the general objectives of the intervention were defined on the basis of the logic model (refer to the Intervention Framework section), the specific learning objectives for the P4PS program on the core topics of patient safety, PFAC engagement, and communication (Textbox 1) were defined based on the taxonomy proposed by Bloom et al [44] and its later revision by Krathwohl [45].

Textbox 1. **Learning objectives of the Partners for Patient Safety program for each topic covered.**

- Patient safety (PS)
  - Understand the fundamentals of PS and quality and risk management as well as recognize their relevance in health care.
  - Analyze examples of errors in health care and complex factors influencing PS and understand different measures to reduce errors (eg, standardization of processes and reporting systems).
  - Apply legal principles in the context of PS in health care.
- Patient and family advisory council (PFAC) engagement
  - Analyze the different levels of engagement of patients and their representatives and the characteristics of these levels.
  - Understand examples of the engagement of PFACs and reflect on their potential impact on the quality and safety of care.
  - Analyze their own understanding of their role, and that of the PFAC as a whole, and reflect on internal and external responsibilities.
  - Adopt and analyze different perspectives (PFAC, health care representatives, and administration) to develop a deeper understanding of the challenges and needs of the other groups.
  - Identify and evaluate at least 3 specific misunderstandings or barriers to collaboration.
  - Analyze possible activities and tasks to engage the PFAC in quality and safety of care in the health care organization.
  - Using methods learned, jointly formulate realistic and measurable goals for collaboration between the PFAC and the health care organization.
- Communication
  - Understand the importance of open and appropriate communication for collaboration in health care contexts.
  - Understand basic communication models and apply them specifically in different contexts.
  - Apply supportive models for decision-making and goal setting in collaborative settings.

#### Intervention Development Group and Supportive Consultations

A total of 5 patient safety researchers, consisting of psychologists, health scientists, and a physician, designed and developed the P4PS program. The research team received support from local institutional services (center for human resources development, university computer center, and faculty members), who provided technical and pedagogical advice regarding the digital realization, organization, and structure of the intervention. Finally, the P4PS program was informally reviewed by an external patient representative, whose user-oriented feedback was incorporated iteratively.

## Results

In this section, we primarily describe the P4PS program in detail and complement this with descriptive information on program use (module completion and use duration) and participant feedback.

### Content and Format of the Intervention

On the basis of key educational needs identified in preceding needs and requirements analysis and the literature review, the P4PS program addressed three core topics: (1) fundamentals of patient safety, (2) engagement of PFACs, and (3) communication and collaborative goal setting.

**Figure 2.**
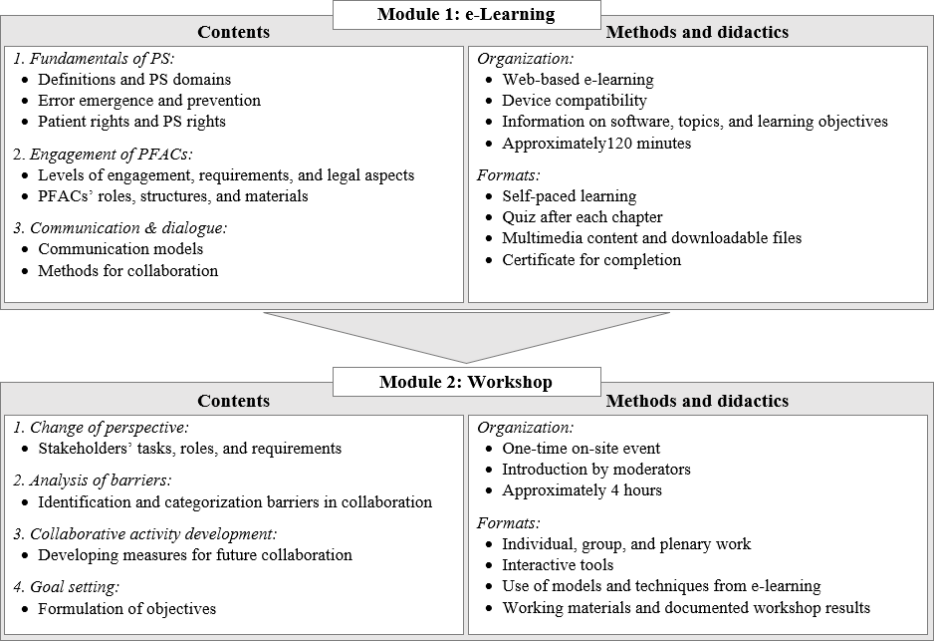
Modules of the Partners for Patient Safety program. PS: patient safety; PFAC: patient and family advisory council.

The P4PS program comprised two major modules: (1) an e-learning module, followed by (2) an on-site workshop module. This structure allows for a combination of passive and active learning approaches and enables participants to receive information as well as apply it in real-world examples. Figure 2 shows the contents, methods, and didactics of the 2 modules of the P4PS program. Details on the e-learning module and workshop module are presented in the following sections.

### e-Learning (Module 1)

#### Topics and Key Content

The e-learning module was designed based on the objective and core topics developed for the P4PS program (refer to the Content and Format of the Intervention section) with three chapters: (1) fundamentals of patient safety, (2) engagement of PFACs, and (3) communication and dialogue.

The first chapter covered definitions of patient safety and adverse events, key patient safety domains, the significance of the topic (eg, adverse event rates and mismanaged conditions), and the sociotechnical complexities that affect patient safety and contribute to errors. Learners were informed about patients’ rights (eg, World Health Organization Patient Safety Rights Charter) and methods for identifying and reducing errors (eg, Swiss cheese model, standardization, hygiene measures, and critical incident reporting).

The second chapter captured engagement of PFACs and provided basic information on patient engagement (ie, levels of engagement, legal requirements, and practical examples of positive influence of PFAC engagement). Learners were presented with videos and various downloadable content covering internal role understanding, examples of high-functioning PFACs organization and structure, and other materials (eg, meeting minutes, PFAC charter, and orientation manual for new members).

The third chapter focused on communication and dialogue for patient safety and included the Speak Up initiative [46] and communication models that fostered respectful dialogue (eg, 4-side model, nonviolent communication, and sandwich method [47-49]). To improve collaboration between PFACs and health care facilities, 2 frameworks were introduced: the facts, options, risks and benefits, decision, execution, and check (FORDEC) decision-making method [50] and the specific, measurable, attainable, realistic, and time-bound (SMART) goal-setting framework [51]. The FORDEC method was selected due to its potential for team decision support and preplanning of risks and failures in complex situations [52,53]. The SMART method is an established approach that has been successfully applied in various disciplines, including medical education [54-57].

The e-learning module concluded with a practice example that demonstrated how a hospital addressed a patient safety issue related to an error, linking it to the course content. More specifically, in this example, a fictitious PFAC collaborated with hospital management to reduce readmission rates. To this end, they set common goals and design strategies to achieve these goals using the SMART and FORDEC methods, which were introduced in earlier chapters of the e-learning module. The developed strategies included informing patients about the Speak Up initiative and providing them with a logbook to record medication changes, questions, and contact information after discharge. This logbook is made available to learners as a downloadable document.

The e-learning module provided various didactic elements, allowing learners to choose suitable options for obtaining information. Options included audio texts, labeled graphics with extra content, and short videos. Learners also engaged with diagrams, graphs, matching exercises, and scenario-based tasks. The intensity of the content delivered increased over the course of the e-learning module, such that basic information on patient safety was followed by in-depth information on strategies, activities, and tasks to promote the engagement of PFACs in patient safety in health care organizations. Learners received downloadable files with information, checklists, and templates for application to their PFAC. Each topic concluded with a short quiz intended to reinforce learning.

We used Rise 360 software (Articulate Global, LLC) to create the e-learning module. Embedded, self-created videos were generated using Microsoft PowerPoint and edited in the DaVinci Resolve editing program (Blackmagic Design Pty Ltd). Audio tracks were generated using the ElevenLabs’ artificial intelligence Text to Speech software. The final e-learning module [58] has been made available on the home page of the Institute for Patient Safety.

#### Didactic Approach, Design, and Procedure

Prior to the introduction of the first chapter, learners received instructions on software management, an overview of topics, learning objectives (Textbox 1), and information on taking breaks.

The e-learning module was designed to be flexible and completed by each learner independently at their own pace. It was accessible via a link and was to be completed before the workshop module (module 2) to aid knowledge retention. It worked on any device (PC, smartphone, tablet, or laptop) with a stable internet connection. A larger device was recommended for better visibility. This module had a duration of approximately 120 minutes.

### Workshop (Module 2)

#### Topics and Key Content

The activities carried out in the workshop module were based on completion of the previous e-learning module (module 1). The workshop module addressed the individual situation of each PFAC and health care organization as well as the respective circumstances. While the e-learning module provided a theoretical foundation, the workshop module focused on translation and contextualization to locally promote mutual communication and collaborative goal development.

On the basis of the formulated learning objectives (Textbox 1), 4 different activities were performed by the participants during the workshop module:

1. A change of perspective, aiming to strengthen learners’ understanding of the complexity of tasks and requirements of the different stakeholders and their individual functions in the care process
2. An identification and categorization of existing barriers and misunderstandings in the previous collaboration between the PFAC and the health care organization
3. The identification or refinement of activities to promote future collaboration
4. The collaborative formulation of concrete objectives for these activities

#### Didactic Approach, Design, and Procedure

In the beginning, the moderators introduced the topic with a short presentation outlining the learning objectives (Textbox 1). Learners were then informed about the course of the workshop module and presented reminders on the preceding e-learning module (eg, communication techniques).

All tasks were performed using different methodological approaches (eg, individual assignments, small or whole group tasks, discussions, and short presentations) and different media (eg, interactive queries, working papers, handouts, and flipcharts). Learners were encouraged by the moderators to apply the communication techniques and models presented in the e-learning module (refer to the e-Learning section). Collaborative development and statement or refinements of objectives were carried out by the learners using the FORDEC and SMART methods, learned in the e-learning module (refer to the e-Learning section). Learners receive additional handouts and worksheets for this purpose. A tabular overview of the workshop activities, methodology, and scheduling can be found in Multimedia Appendix 3.

The results of the workshop modules were documented and shared with participants in a summary. The workshop modules were organized as one-time and on-site events that lasted approximately 4 hours.

### Program Use and Participant Feedback

A total of 36 participants (PFAC members and health care representatives) engaged with the P4PS program. Of these 36 participants, 33 (92%) completed module 1 (ie, e-learning module) and 26 (72%) took part in module 2 (ie, workshop module). Completion rates across the 8 chapters of the e-learning module ranged from 94% (31/33) to 100% (36/36). On average, participants spent 140.5 (SD 65.3) minutes completing the e-learning module. Regarding feasibility and preliminary effectiveness, the program was generally described as usable, user-friendly, relevant, and effective for building competencies in patient safety and communication. Challenges included technical skills, lay language, and the degree of interactivity, which did not, however, hinder completion. Details of the formal evaluation, including outcomes on feasibility and preliminary effectiveness, are reported in a separate publication.

## Discussion

### Principal Findings

Responding to the call for increased engagement of patients and their representatives in patient safety at the organizational level and associated challenges of inadequate programs to promote collaboration, we developed the P4PS program on patient safety and communication for members of PFACs and health care representatives. To the best of our knowledge, this is one of the first codeveloped training initiatives specifically designed to build PFAC capacity in patient safety–related care issues. Moreover, this P4PS program can be a novel step toward institutionalizing collaborative structures. Evaluating its feasibility and effectiveness is crucial to understand how such innovations can be sustainably implemented and scaled; however, to provide more detailed reporting, this study focuses on the systematic development of the intervention itself, while the evaluation is reported in detail elsewhere.

In the course of the program’s development, we sought to establish conceptual and methodological rigor by drawing on established frameworks such as the logic model and the taxonomy proposed by Bloom et al [44], as well as by engaging PFAC members and stakeholders. The use of the logic model [59] is expected to support targeted planning based on existing resources and to facilitate the program’s implementation, replication, and translation into practice. In turn, the taxonomy proposed by Bloom [60] supports the gradual development of cognitive skills and continuous learning progress during the P4PS program. Additionally, the engagement of PFAC members for intervention development ensured a needs-based intervention and suitable content for knowledge transfer. Through our comprehensive reporting, which is often lacking in similar initiatives [20,61], we enhance the potential for replication in other health care settings and adaptation to different contexts.

Concerning its future implementation, our intervention development addressed several challenges that often limit sustainable success of such patient engagement measures in practice. For instance, to ensure its relevance to the national context (ie, Germany), we screened the international literature [37,38,41,42] and finalized key content in consultation with a patient representative. Furthermore, we incorporated findings from a previous needs and requirements analysis [19], a scoping review, and an earlier German intervention study on skills development into the intervention [39]. Despite the thorough planning stage, we acknowledge that this approach requires significant time and resource investment, which can be a barrier for feasibility across health care organizations [62]. As involvement of stakeholders and recruiting participants can be challenging, health care organizations should proactively communicate the relevance of the program in dialogue with PFACs. Visibility and attractiveness can be increased by monitoring participation data and linking it to improvements in patient safety initiatives [63].

Another factor affecting implementation is the background of PFAC members, which can vary widely across organizations. Studies show that a lack of motivation, feedback, or knowledge [64,65] as well as technical and time-related hurdles inhibit engagement [66]. Educational programs integrated into the onboarding process can help establish a shared knowledge base. Support personnel from the health care sector may facilitate access to resources [27], while incentives such as certificates or perks for long-term project participation may strengthen willingness to engage. Optional e-learning modules that allow for tailored content selection may address the varying needs among stakeholders of different PFACs [67]. However, feasibility must be maintained to ensure accessibility and focus. Consideration of such context-specific factors will also be relevant in evaluating the program’s transferability.

Future evaluation of the P4PS program plays a decisive role in further development and improvement. As one of the first initiatives of its kind in Germany, the P4PS program builds on specific needs and fulfills the specific purpose of training PFACs, which requires a detailed description and has led us to address these needs pragmatically. Future program testing for feasibility and effectiveness using a mixed methods approach will allow identification of strengths and weaknesses.

It can be assumed that the P4PS program will only reach its full potential if favorable policy, structural, and procedural changes are made in the health care system at the same time [68-70]. Future research should pursue measures to implement such programs, especially depending on different underlying organizational contexts [71]. A comparable intervention, if properly implemented, has the potential to initiate appropriate changes to strengthen the voice of patients and their advocates in promoting safe care systems.

### Conclusions

This study provides a comprehensive account of the systematic development of the P4PS program for PFACs and health care representatives. Building on our previous efforts to identify PFACs’ needs and relevant evidence, the program has the potential to be used as a viable measure to promote PFACs’ engagement in fostering patient safety in health care organizations. As the landscape of health care services evolves quickly, continued commitment to empowering patients and patient advocates through needs-oriented and pragmatic measures is essential in fostering a culture of safety and mutual collaboration.

## Supplementary material

10.2196/79286 Multimedia Appendix 1 Complete logic model for the intervention description and potential evaluation.

10.2196/79286 Multimedia Appendix 2 Summary of key findings from the main sources that informed intervention development.

10.2196/79286 Multimedia Appendix 3 Workshop activities, methodology, and schedule.

10.2196/79286 Checklist 1 TIDieR checklist.

10.2196/79286 Checklist 2 GUIDED checklist.

## Abbreviations

FORDEC: facts, options, risks and benefits, decision, execution, and check
GUIDED: Guidance for the Reporting of Intervention Development
P4PS: Partners for Patient Safety
PFAC: patient and family advisory council
SMART: specific, measurable, attainable, realistic, and time-bound
TIDieR: Template for Intervention Description and Replication
