# Supplementary material for: A Blended Educational Program to Promote Dialogue on Patient Safety Between Patient and Family Advisory Councils and Health Care Organizations: Codevelopment Study
Source: JMIR Form Res. 2025 Nov 24;9:e79286. doi: 10.2196/79286 (PMC12643403; doi:10.2196/79286)
Supplement: Multimedia Appendix 1 [file formative-v9-e79286-s001.pdf]

Multimedia Appendix 1

Complete logic model for the intervention description and potential evaluation.

Level	Description	Key Assumptions	Objectively Identifiable Indicators	Means of Verification
Goal	Improving PS and quality of care at an organizational level by engaging patients, their families and representatives in health care	• Increased engagement of PFACs in health care in Germany improves PS<br>• PS is impaired by inadequate knowledge of PS and communication	• Improvement of PS metrics, e.g. number of adverse events per year in areas/stations with PFAC connection in relation to comparable stations without PFACs (before implementation of PS measures)	• Annual hospital statistics from hospital monitoring systems (CIRS)<br>• Reports and analyses on PS
Results	• Increased engagement of PFACs in decisions on quality, care processes and PS issues<br>• Sustainable networks and partnerships between PFACs and HCO	• PFAC HCR are interested in promoting the engagement of PFAC in PS topics<br>• HCO and PFACs know how to measure engagement<br>• HCO and PFACs know how PS-relevant topics can be evaluated	• Increased safety culture<br>• Number of initiatives to strategically promote PS<br>• Number and type of sustainable networks and partnerships<br>• Long-term implementation of documented objectives (see description of outputs)	• Tool to evaluate safety culture (e.g. HSOPSC 2.0)<br>• Tool to evaluate PE (e.g PPEET)<br>• Documentation of the initiatives<br>• Network and partnership reports
Objectives	**Increased engagement of PFACs through improved quality and quantity of communication between participating PFACs and HCR on PS topics**	• PFACs need better PS and dialogue skills for gaining a competent voice in health care<br>• Communication and partnership-based dialogue between PFACs and HCR must be expanded	• Number and quality of interactions in the exchange of experiences<br>• Number of participants with expected improvement in quality and quantity of communication between participating PFACs and HCR on PS topics.	• Reports on experience sharing and interactions<br>• Surveys, tools, interviews<br>• Teamwork and PS questionnaire adapted<br>• Speak Up Check questionnaire adapted
Outputs	*Participating PFAC and HCR:*<br>• Understand the necessary fundamentals of PS and can recognize PS-relevant events	*Participating PFAC and HCO:*<br>• Are interested in promoting the engagement of PFAC in PS processes	• Number of persons with completed theoretical program (part 1)	• Participant lists and feedback forms<br>• Evaluation reports

	• Understand the models and strategies of effective communication presented<br>• Understand examples of good engagement of PFACs in care processes<br>• Understand the perspectives of other people involved and explain their activities and functions in the care process<br>• Identify barriers and misunderstandings in communication between PFAC and HCO<br>• Develop new possibilities for future activities and collaboration	• Are interested in discussing and reflecting on cases of (near) harm that have occurred in patient care<br>• Speak openly about communication barriers and PS risks<br>*Also:*<br>• HCO provide opportunities for closer collaboration<br>• Resources are made available to ensure improved collaboration	• Number of people who have completed a workshop (part 2)<br>• Number of trainings / workshops conducted<br>• Number and type of materials used<br>• Increase in knowledge and competence scores in pre- and post-tests<br>• General meeting reports (on the respective outputs)	• Minutes from the workshops incl. defined objectives of the PFACs<br>• Review of learning progress on PS topics, dialogue skills via developed effectiveness items
Activities	• Intervention development based on needs and requirements as well as international literature<br>*PFAC and HCR:*<br>• Are trained in PS content, examples of PFAC engagement and communication skills<br>• Are presented with new communication activities to foster dialogue<br>• Are given the opportunity to systematically apply the options presented to their own situation<br>• Receive support to promote common goals and future collaboration	• PFAC and HCR take part in surveys and interventions<br>• PFACs share specific needs and requirements on PS and dialogue topics<br>• Interventions developed are effective in promoting PS and communication skills<br>• Interventions developed are effective in promoting collaboration	• Number and type of information materials, tools, training and workshop formats produced<br>• Number and type of workshops and training courses held<br>• Total number of participants/per advisory board<br>• Number of interviews and surveys conducted	• Description of the intervention formats and content<br>• Reports and materials from the training courses, event reports<br>• Participation lists<br>• Research reports and data analyses<br>• Interviews, surveys
Inputs	• Interview guide for needs and requirements assessment<br>• Online questionnaire to prioritize content for intervention.<br>• Literature research on comparable, already existing programs<br>• Other infrastructure and resources, e.g.:<br>a. Funding	*Responsible persons provide:*<br>• Resources to develop the intervention<br>• Materials for the participants<br>*Generally existing:*<br>• Adequate funding<br>• Availability of PFACs and HCR.	• Approved funding<br>• Number and qualifications of project members<br>• Availability and condition of resources<br>• Number of valid declarations of consent<br>• Ethics vote	• Financing agreement<br>• Contracts of the project members<br>• Inventory lists<br>• Ethics vote

	b. Personnel expertise of study team<br>c. Support with and resources for the development of educational programs	• Willingness and consent of PFACs and HCR to participate.<br>• No ethical concerns about the project.	

Levels, descriptions, key assumptions, objectively identifiable indicators and means of verification of the logic model. Vertical logic works backwards from *inputs* to *goals*, to establish a conceptual framework and operational definitions for each level. Horizontal logic explains the requirements needed to achieve each level. PS – patient safety, PFAC – patient and family advisory council, CIRS – critical incident reporting system, HCO – healthcare organization, HCR – healthcare representative, HSOPSC 2.0 – Hospital Survey on Patient Safety Culture (second version), PE – patient engagement, PPEET – Public and Patient Engagement Evaluation Tool
