# Supplementary material for: A Blended Educational Program to Promote Dialogue on Patient Safety Between Patient and Family Advisory Councils and Health Care Organizations: Codevelopment Study
Source: JMIR Form Res. 2025 Nov 24;9:e79286. doi: 10.2196/79286 (PMC12643403; doi:10.2196/79286)
Supplement: Multimedia Appendix 2 [file formative-v9-e79286-s002.pdf]

Multimedia Appendix 2

Summary of key findings from the main sources that informed intervention development

1. *Brust L, Blum Y, Weigl M. Promoting Patient Safety Through Patient Engagement at the Organisational Level: A Delphi-Based Needs Assessment Among Patient and Family Advisory Councils. Health Expectations. 2025;28:e70319. doi: 10.1111/hex.70319*

Nineteen stakeholders from six patient and family advisory councils (PFACs) across German university hospitals participated in this study. Findings indicate that PFACs' levels of engagement and rights of participation vary substantially across topics and institutions. While collaboration is perceived as respectful, the actual influence of PFACs on decision-making remains limited and often unclear. Participants expressed a strong desire for deeper access to clinic-specific information and processes to enable meaningful contributions.

Patient safety has thus far not been an explicit focus within PFAC activities. Nonetheless, participants emphasized their willingness to engage more actively in patient safety, provided that they gain access to relevant data and processes. PFACs envisioned potential roles ranging from patient support and community outreach to active participation in quality management and governance structures. However, a prerequisite for such engagement is the clear definition and communication of PFACs' roles, responsibilities, and boundaries.

A Delphi process identified ten relevant competency domains for future educational interventions: five related to healthcare safety and quality (e.g., fundamentals of patient safety, hygiene management, legal basis of patient engagement, quality and risk management) and five concerning communication (e.g., clear and respectful manners, plain communication, constructive feedback). PFAC stakeholders highlighted limited knowledge and skills in these domains, underlining the necessity of targeted training. Preferred formats include modularized interventions, combining short theoretical elements (≤120 minutes, often online) with longer practical workshops (4h to a full day, preferably in person for networking).

2. *Rramani Dervishi Q, Blum Y, Brust L, et al. Engaging patient and family advisory councils (PFACs) in patient safety in healthcare organizations: A rapid scoping review. Under Review. 2025.*

The review identified 13 educational resources on PFAC engagement in patient safety, comprising six peer-reviewed studies and seven gray literature sources, predominantly from the USA and Canada. Formats ranged from workshops, courses, and coaching sessions to handbooks, guides, and hands-on tools, with most resources combining multiple elements. Patient safety-related content frequently included definitions of core concepts, roles of advisors, and examples of engagement projects; fewer resources addressed specific topics such as error prevention, infection control, or legal and ethical issues. Auxiliary topics such as communication, storytelling, orientation to healthcare systems, and confidentiality training were also common.

Educational approaches combined passive (e.g., readings, presentations) and active learning (e.g., discussions, exercises), delivered in in-person, virtual, or hybrid modes, and targeted both individual and group settings. Reported durations varied from short sessions (<1h) to multi-hour workshops. Resources were typically developed by interprofessional groups and delivered by clinicians, managers, facilitators, or experienced advisors. Evaluations were limited, with few studies assessing satisfaction, feasibility, or practice-related outcomes, and only one gray literature source reporting usability testing.
