# Supplementary material for: A Blended Educational Program to Promote Dialogue on Patient Safety Between Patient and Family Advisory Councils and Health Care Organizations: Codevelopment Study
Source: JMIR Form Res. 2025 Nov 24;9:e79286. doi: 10.2196/79286 (PMC12643403; doi:10.2196/79286)
Supplement: Multimedia Appendix 3 [file formative-v9-e79286-s003.pdf]

Multimedia Appendix 3

Workshop activities, methodology, and scheduling.

**Type of event:**	Workshop
**Session topic:**	Patient safety and communication to promote engagement of patient and family advisory councils (PFAC)
**Session goals:**	1. Participants can identify and reflect on the challenges of and expectations for different roles in healthcare and develop a deeper understanding of the different perspectives and functions.<br>2. Participants have an understanding of communication and role-related problems and can identify and name barriers to cooperation between the PFAC and healthcare organization.<br>3. Participants can apply the FORDEC method to develop specific activities to promote PFAC engagement.<br>4. Participants can apply the SMART method to formulate common goals of the PFAC and the healthcare organization.
**Learning objectives:**	1. Adopt and analyze different perspectives (PFAC, healthcare representatives, administration) in order to develop a deeper understanding of the challenges and needs of the other group.<br>2. Identify and evaluate at least three specific misunderstandings or barriers to collaboration.<br>3. Analyze possible activities and tasks to engage the PFAC in quality and safety of care in the healthcare organization.<br>4. Using methods learned, jointly formulate realistic and measurable goals for collaboration between the PFAC and the healthcare organization.
**Duration:**	4 hours

Topic	Total Time	Topic Detail	Detail Time	Learning Objective	Method	Procedure	Learning Objective Check	Media/ Material	Remarks
**Introduction**	20 Min	Welcome & Organisation	3 Min		Presentation	Brief welcome and introduction of IfPS and moderators.			
		Introduction & Icebreaker	10 Min		Chain Reaction	The question "What motivated you to participate today?" is displayed. The participants state their name, time on the PFAC, role/function, and answers the question.		Mobile phone with stopwatch	
		Agenda	3 Min		Presentation	The workshop agenda is presented to the participants.		PowerPoint	
		Learning Objectives	3 Min		Presentation	The learning objectives of the workshop are presented to the participants (including explanation of why they were created this way); any arising questions are addressed.		PowerPoint	
		Results of Needs Analysis	4 Min		Presentation	Brief presentation of the key results of the needs assessment to provide a thematic transition.			
**Change of Perspective**	40 Min	Explanation	3 Min	1	Presentation	The learning objective and procedure of the next activity are presented by the moderators. Refer to case study from the e-learning.		PowerPoint	
		Clarification of Guiding Questions	4 Min	1	Presentation	The moderators present the guiding questions for the activity. The participants should use these guiding questions to shape the subsequent discussion. Guiding questions: - What tasks and functions does this person fulfill regarding quality and safety of care? - What expectations are placed on the person? - What (false) assumptions does the person feel exposed to in their professional context?	The goal is to clarify and objectify possible assumptions about the respective activities, tasks, and functions through the role change. This should strengthen the understanding of the respective other groups of people.	PowerPoint	

Topic	Total Time	Topic Detail	Detail Time	Learning Objective	Method	Procedure	Learning Objective Check	Media/ Material	Remarks
		Group Assignment	2 Min	1	Presentation	Group assignment: Each group consists of members of the PFAC and health care representatives who take on the other's perspective. If not enough health care representatives are present, roles are taken over by people involved in the process.		"Name tags" for participants to attach, indicating the role they should assume	
		Discussion	20 Min	1	Group Discussion	Discussion within the small groups based on the guiding questions. - What new insights about the different points of view have emerged?			
		Reflection	10 Min	1	Group Discussion	Reflection of the activity together with all members, each briefly states their statement; results are written down	At least 3 misunderstandings about views on respective other roles can be identified.	Flipchart	
BREAK	15 Min								
**Identifying Barriers**	35 Min	Explanation	5 Min	2	Presentation	The learning objective and procedure of the next activity are presented by moderators		PowerPoint	
		Identification (Individual)	10 Min	2	Individual Work	Each person individually notes barriers and grievances in the previous cooperation in Mentimeter. Topic areas: Communication, responsibilities / contact persons / understanding of roles, general tasks of the advisory board, competences		Mentimeter	
		Identification (Group)	10 Min	2	Group Discussion & Presentation	The individually collected barriers and misunderstandings are compiled via Mentimeter. Then, the individual points are assigned to upper categories via flip chart. Then, the upper categories are entered back into Mentimeter.		Flipchart, PowerPoint, Mentimeter	

Topic	Total Time	Topic Detail	Detail Time	Learning Objective	Method	Procedure	Learning Objective Check	Media/ Material	Remarks
		Evaluation	10 Min	2	Group Discussion	The created upper categories of barriers and grievances are evaluated by the participants via Mentimeter. Each participant gets three votes and evaluates the corresponding barriers according to their personal priority.	3 main barriers can be identified.	Flipchart, Mentimeter	
BREAK	5 Min								
**Engagement of PFAC**	45 Min	Explanation	5 Min	3	Presentation	The learning objective and procedure of the next activity are presented by moderators. Reference to case study from the e-learning.		PowerPoint, FORDEC method handout	
		Presentation of Examples	10 Min	3	Presentation	Moderators present successful examples of activities for possible engagement of PFACs (see e-learning, preferably with as close a reference to the previously mentioned barriers as possible). The FORDEC method is recalled.		PowerPoint	
		Brainstorming	15 Min	3	Group Work	Division into small groups. Each group develops ideas for specific activities that could promote the engagement of the PFAC in certain tasks of care, using the FORDEC method (ideas can refer to the barriers from activity 2 or be freely chosen).		Paper, pens, FORDEC paper DIN-A-2	
		Presentation & Discussion	15 Min	3	Group Discussion	The groups come together and present their results, the results are collected and discussed for their feasibility.	At least 4 activities developed with the FORDEC method can be identified.	Flipchart, PowerPoint	
BREAK	15 Min								
**Common Goals**	35 Min	Explanation	5 Min	4	Presentation	Learning objective and procedure of the next activity are presented by moderators, SMART method is recalled		PowerPoint, SMART method handout	

Topic	Total Time	Topic Detail	Detail Time	Learning Objective	Method	Procedure	Learning Objective Check	Media/ Material	Remarks
		Goal Setting	15 Min	4	Group Discussion	Applying the SMART method, goals for the previously collected specific activities for PFAC engagement are jointly formulated in the large group + possibilities for evaluating the achievement of goals are determined.		Flipchart, PowerPoint	
		Goal Discussion	15 Min	4	Presentation	The formulated goals are repeated and checked for consensual agreement by the participants.	3 main goals formulated according to SMART can be identified.	Flipchart, PowerPoint	
**Conclusion**	30 Min	Organization	15 Min			On-site implementation of the online questionnaire (post evaluation) + find participants for interviews.			
		Feedback	15 Min		Open Feedback Round	Each participant expresses in two sentences: Impressions and insights from the workshop, summary of the most important results and next steps.		PowerPoint	

IfPS – Institute for Patient Safety; PFAC – Patient and Family Advisory Council; FORDEC – Facts, Options, Risks, Decisions, Executions, Check; SMART – Specific, Measurable, Attainable, Realistic, Timely.
