# Supplementary material for: A Blended Educational Program to Promote Dialogue on Patient Safety Between Patient and Family Advisory Councils and Health Care Organizations: Codevelopment Study
Source: JMIR Form Res. 2025 Nov 24;9:e79286. doi: 10.2196/79286 (PMC12643403; doi:10.2196/79286)
Supplement: Checklist 1 [file formative-v9-e79286-s004.pdf]

Appendix A

Table 1 Intervention description using the ‘Template for Intervention Description and Replication’ (TIDieR) checklist[1].

Item number	Item	Where located	
		Primary paper (page or appendix number)	Other (details)
**1.**	**Brief name:** Provide the name or phrase that describes the intervention.	**3**	
**2.**	**Why:** Describe any rationale, theory or goal of the elements essential to the intervention.	**2-3**	
**3.**	**What (Materials):** Describe any physical or informational materials used in the intervention, including those provided to participants or used in intervention delivery or in training of intervention providers.	**11-15**	
**4.**	**What (Procedures):** Describe each of the procedures, activities, and/or processes used in the intervention, including any enabling or supporting activities.	**11-15**	
**5.**	**Who Provided:** For each category of intervention provider (e.g. psychologist, nursing assistant), describe their expertise, background and any specific training given.	**9***	
**6.**	**How:** Describe the modes of delivery (e.g. face-to-face or by some other mechanism, such as internet or telephone) of the intervention and whether it was provided individually or in a group.	**11-12,14-15**	
**7.**	**Where:** Describe the type(s) of location(s) where the intervention occurred, including any necessary infrastructure or relevant features.	**12-13***	

**8.**	**When and how much:** Describe the number of times the intervention was delivered and over what period of time including the number of sessions, their schedule, and their duration, intensity or dose.	**12-13***	
**9.**	**Tailoring:** If the intervention was planned to be personalised, titrated or adapted, then describe what, why, when, and how.	**4-9**	
**10.**	**Modifications and how well:** If the intervention was modified during the course of the study, describe the changes (what, why, when, and how).	**16****	
**11.**	**How well (Planned):** If intervention adherence or fidelity was assessed, describe how and by whom, and if any strategies were used to maintain or improve fidelity, describe them.	**16****	
**12.**	**How well (Actual):** If intervention adherence or fidelity was assessed, describe the extent to which the intervention was delivered as planned.	**16****	

* The intervention had not yet been carried out at the time the manuscript was written. The dates given describe the planned procedure.
** Evaluation of the intervention with regard to feasibility using the described indicators is planned.

References

1. Hoffmann TC, Glasziou PP, Boutron I, et al. Better reporting of interventions: template for intervention description and replication (TIDieR) checklist and guide. *BMJ*. 2014;348:g1687. doi:10.1136/bmj.g1687
